# The double-edged mechanism of organizational AI adoption on employee creativity

**DOI:** 10.3389/fpsyg.2026.1921889

**Published:** 2026-09-01

**Authors:** Rui Mao, Leilei Zhou

**Affiliations:** 1 School of Business Administration, Guizhou University of Finance and Economics, Guiyang, China; 2 Chengdu Normal University, Chengdu, China; 3 School of Management, Guizhou University, Guiyang, China

**Keywords:** AI replacement anxiety, AI self-efficacy, creativity, empowering leadership, organizational AI adoption

## Abstract

**Introduction:**

As artificial intelligence (AI) becomes increasingly integrated into organizational management, understanding its association with employee creativity has become an important research issue.

**Methods:**

Drawing on self-determination theory, this study examines the two psychological pathways linking organizational AI adoption to employee creativity and investigates the moderating role of empowering leadership. A two-wave survey was conducted among 400 employees from Chinese organizations. Hierarchical regression analyses and bootstrapping procedures using the PROCESS macro were employed to test the proposed moderated mediation model.

**Results:**

Organizational AI adoption is negatively associated with employee creativity through AI replacement anxiety, while it is positively associated with employee creativity through AI self-efficacy. These two significant indirect effects operated in opposite directions and were similar in magnitude, thereby largely offsetting each other and indicating a countervailing mediation pattern. Furthermore, empowering leadership weakened the relationship between organizational AI adoption and AI replacement anxiety while strengthening its relationship with AI self-efficacy.

**Discussion:**

This study extends the literature on the consequences of organizational AI adoption by identifying its countervailing associations with employee creativity from the perspective of self-determination theory. The findings also highlight the critical role of empowering leadership in weakening the negative pathway and strengthening the positive pathway associated with AI adoption, offering practical implications for organizations seeking to foster employee creativity in the AI era.

## 1 Introduction

With the rapid advance of a new digital technological revolution represented by generative artificial intelligence, AI is widely integrated into organizational and operational scenarios, such as personnel recruitment, performance appraisal, customer service, operations management, risk prediction, and strategic decision-making, in order to improve organizational efficiency and competitive advantage, becoming an important driving force for enterprise digital transformation (Csaszar et al., 2024; Jiang et al., 2025; Kelan, 2024; Tursunbayeva and Chalutz-Ben Gal, 2024). Such organizational AI adoption has fundamentally disrupted traditional work methods and task structures, shifting employees’ work environment from predominantly human–human interaction toward increasingly complex forms of human–AI collaboration (Seeber et al., 2020). In this context, employees not only need to adapt to the changes in tasks and work styles brought about by AI, but also re-evaluate their own skills, professional roles, and their value in human–AI collaboration (Tang et al., 2023; Bankins et al., 2024). Understanding how employees perceive and respond to organizational AI adoption has therefore become an important issue in organizational behavior research.

In this study, organizational AI adoption refers to employees’ overall perceptions of the extent to which their organization has introduced and integrated AI technologies into work processes and job-related activities. However, AI technology itself cannot be directly transformed into organizational value. Its outputs still need to be understood, judged, and further processed by employees in relation to their specific work roles before they can become truly novel and practical solutions (Ma et al., 2024). From the perspective of the creativity formation process, employee creativity relies on cognitive activities such as problem identification, information acquisition, knowledge recombination, and solution generation, and organizational AI adoption embeds and changes these key processes, which may affect the way employees acquire information, integrate knowledge, and form solutions (Tolkamp et al., 2022; Csaszar et al., 2024). However, existing studies on organizational AI adoption have paid more attention to issues such as employee well-being and retention intention, while still giving insufficient attention to the generative outcome of how employees embed AI into work processes and transform it into creative contributions (Valtonen et al., 2025; Jiang et al., 2026). Based on this, this study regards employee creativity as an important outcome variable for understanding the employee-level effects of organizational AI adoption, and aims to explore how organizational AI adoption influences employee creativity, thereby enriching related research on organizational AI adoption.

Prior studies have mostly explained the effects of new technologies on employee behavior from the perspectives of the job demands–resources model or conservation of resources theory, emphasizing the processes of resource gain or resource loss brought about by technology (Chuang et al., 2025; Liu et al., 2025). Nevertheless, these studies often examine the threatening and enabling consequences of AI separately, making it difficult to explain why the same organizational AI adoption context may be associated with opposing psychological processes and why its overall relationship with employee creativity may appear weak or inconsistent. Meanwhile, employee creativity depends heavily on the activation of deep intrinsic motivation and the satisfaction of basic psychological needs (Ye et al., 2025). Self-determination theory provides an appropriate framework for understanding these divergent responses. According to this theory, organizational AI adoption may shape employees’ autonomy and competence needs by altering how they perceive their abilities, work roles, and career prospects (Hermann et al., 2025; Ryan and Deci, 2020). When organizational AI adoption frustrates employees’ needs for autonomy and competence by threatening their jobs, skills, or occupational value, it may trigger AI replacement anxiety and subsequently inhibit employee creativity (Kim and Kim, 2024). Conversely, when organizational AI adoption satisfies these needs by supporting learning, capability development, and work control, it may strengthen AI self-efficacy and, in turn, promote employee creativity (Jeong and Jeong, 2024).

**Figure 1:**
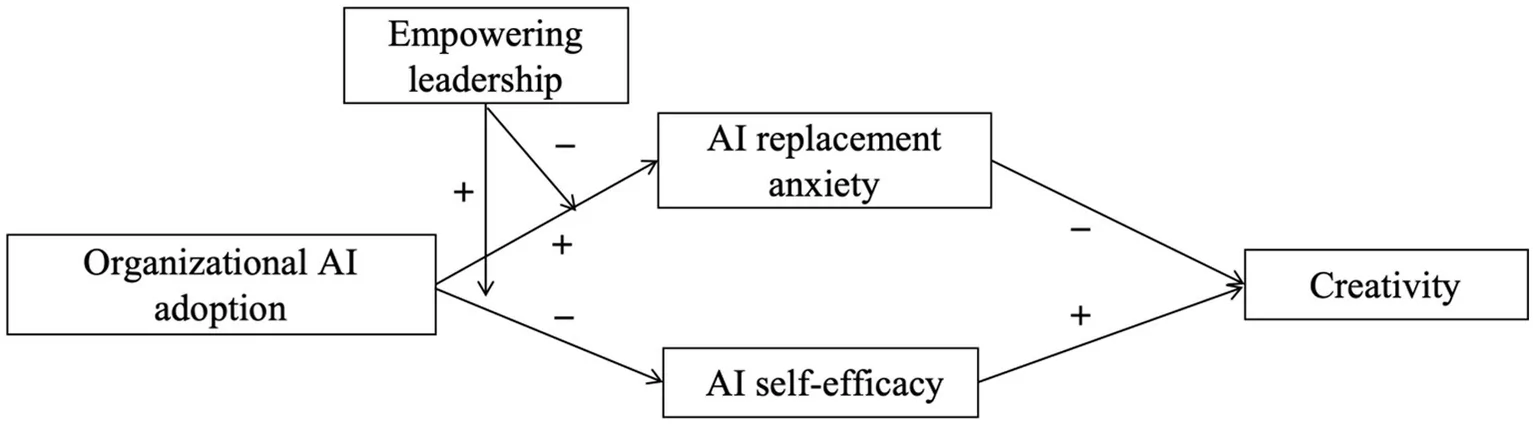
Theoretical framework.

Existing studies on the boundary conditions of technology adoption or organizational AI adoption have mainly focused on employee characteristics or technological features, emphasizing how factors such as technology acceptance and algorithmic transparency affect employees’ acceptance and use of technology (Cai et al., 2026; Zhang et al., 2026). However, relatively limited attention has been paid to the role of immediate leadership in shaping employees’ psychological responses to organizational AI adoption. Organizational AI adoption is typically initiated as an organization–level technological decision, whereas immediate supervisors serve as an important link between organization-level AI initiatives and employee–level psychological responses (Makarius et al., 2020; Wang et al., 2025). Self-determination theory suggests that external social contexts shape employees’ psychological responses to technological change and work pressure by influencing the satisfaction or frustration of their basic psychological needs, including autonomy, competence, and relatedness, thereby affecting their motivation and organizational behavior (Ryan and Deci, 2020; Gagné et al., 2022). Empowering leaders can enhance employees’ sense of autonomy and competence by granting them autonomy, encouraging participation in decision-making, and providing support (Cheong et al., 2019). In a high empowering leadership context, by supporting employees’ autonomy and competence, empowering leadership may weaken the association between organizational AI adoption and AI replacement anxiety while strengthening its association with AI self-efficacy (Knevelsrud et al., 2025; Nguyen et al., 2026). Therefore, this study suggests that empowering leadership shapes an organizational environment that supports autonomy and ability, and examines its moderating role in the relationship between organizational AI adoption and employee AI replacement anxiety and AI self-efficacy. The research model is shown in Figure 1.

## 2 Theoretical foundation and research hypotheses

### 2.1 Self-determination theory

Self-determination theory suggests that individuals have an intrinsic motivation to develop, learn, and integrate external experiences, but whether this motivation can be translated into positive behavior depends on the satisfaction of their basic psychological needs (Ryan and Deci, 2020). These needs include autonomy, competence, and relatedness. Autonomy refers to the extent to which individuals feel that their actions are driven by their own volition; competence refers to the belief that they can effectively meet task and environmental demands; and relatedness refers to the sense of connection with others (Slemp et al., 2024). In organizational contexts, the social environment can shape employees’ intrinsic and autonomous motivation by either satisfying or thwarting these basic psychological needs. When employees’ needs for autonomy, competence, and relatedness are satisfied, they are more likely to show higher work engagement, proactive learning, and creative behavior. When these needs are frustrated, they are more likely to experience stress, burnout, alienation, and lower creative performance (Deci et al., 2017; Hagger and McAnally Star, 2026).

In contemporary workplaces, organizational AI adoption has a dual psychological potential: it may undermine employees’ autonomy and competence while also creating conditions that support these needs (Zirar et al., 2023; Gagné et al., 2022). Organizational AI adoption may also lead employees to perceive a decline in the value of their skills and a threat to future job security, hindering the satisfaction of their basic needs, triggering AI replacement anxiety, and suppressing creativity (Kim and Kim, 2024).

### 2.2 The negative pathway linking organizational AI adoption to employee creativity

AI replacement anxiety refers to employees’ feelings of threat arising from concerns that artificial intelligence may replace their jobs or diminish the value of their occupational skills and roles (Brougham and Haar, 2018). According to self-determination theory, when the work environment frustrates employees’ needs for autonomy and competence, they are more likely to experience negative emotions and defensive psychological responses. Therefore, organizational AI adoption may frustrate employees’ basic psychological needs and increase AI replacement anxiety (Gagné et al., 2022). First, organizational AI adoption may strengthen employees’ perception that technology can replace human labor, increasing their career insecurity (Yam et al., 2023). Second, the high efficiency and accuracy of AI systems in data processing and task execution may undermine employees’ confidence in the continued value of their expertise, intensifying concerns about AI-driven replacement (Kim and Kim, 2024; Hermann et al., 2025). Finally, organizational AI adoption is often accompanied by job restructuring and changing skill requirements, and this uncertainty may reduce employees’ sense of control over their work context (Gagné et al., 2022; Cheng et al., 2023). Based on this, we propose:

*H1*: Organizational AI adoption is positively related to AI replacement anxiety.

AI replacement anxiety can be understood as an AI-specific downstream psychological response associated with the frustration of employees’ needs for autonomy and competence (Dong et al., 2025). When employees perceive that AI may reduce their control over work, devalue their existing skills, or threaten their future occupational roles, such need-frustrating experiences may weaken autonomous motivation and, in turn, undermine creativity. First, creativity usually requires a high level of cognitive resources. AI replacement anxiety may lead employees to pay continued attention to whether they can maintain control over their work and preserve the value of their capabilities in an AI-enabled workplace, thereby occupying limited attention and cognitive resources and reducing their psychological capacity for creative thinking (Probst et al., 2020; Gong et al., 2023). Second, the frustration of autonomy and competence may shift employees’ motivational orientation from growth and exploration toward self-protection. Accordingly, employees experiencing stronger AI replacement anxiety may be more likely to adopt defensive behaviors and conservative work strategies to avoid errors and potential losses, reducing their willingness to experiment with unconventional ideas and suppressing creativity (Kim and Kim, 2024). In addition, anxiety may narrow the scope of attention and information processing, limiting cognitive flexibility and reducing employees’ ability to generate novel ideas (Daker et al., 2023). Thus, we propose:

*H2*: AI replacement anxiety is negatively related to employee creativity.

*H3*: AI replacement anxiety mediates the relationship between organizational AI adoption and creativity.

### 2.3 The positive pathway linking organizational AI adoption to employee creativity

AI self-efficacy refers to employees’ confidence in their ability to understand, engage with, and effectively use AI technology in the workplace (Dong et al., 2025). According to self-determination theory, organizational AI adoption may enhance AI self-efficacy by creating a work environment that supports employees’ needs for competence and autonomy. AI self-efficacy is not equivalent to basic psychological need satisfaction; rather, it represents an AI-specific capability belief that may develop when employees feel competent in using AI and experience sufficient autonomy in determining how AI is incorporated into their work (Gagné et al., 2022). First, organizational AI adoption can provide employees with richer information resources, data analysis support, and intelligent feedback, enabling them to gain stronger problem-solving ability when handling complex tasks and strengthening their confidence in using AI (Liu and Mei, 2026). Second, the task restructuring brought by organizational AI adoption can free employees from repetitive and low-value work, allowing them to take the lead in more challenging and innovative tasks, which significantly enhances their AI self-efficacy (Wang et al., 2020). Based on this, the following hypothesis is proposed:

*H4*: Organizational AI adoption is positively related to AI self-efficacy.

AI self-efficacy reflects employees’ confidence in their ability to understand and effectively use AI technologies in their work. It can strengthen employees’ perceived competence and sense of mastery over AI-enabled tasks, and make employees more willing to explore new methods and engage in creative attempts, positively affecting employee creativity (Gagné et al., 2022). Creativity requires employees to believe that they are capable of proposing new ideas, solving complex problems, and dealing with uncertain tasks (Haase et al., 2018). Employees with stronger AI self-efficacy are more confident in using AI for information search, problem diagnosis, solution generation, and idea refinement, which facilitates creative exploration and experimentation (Jeong and Jeong, 2024). Such confidence also makes employees more likely to regard AI as a resource that extends their capabilities rather than as a technology that threatens their role, allowing them to devote greater cognitive effort to divergent thinking and the development of novel solutions (Jia et al., 2024). Based on this, we propose:

*H5*: AI self-efficacy is positively related to employee creativity.

*H6*: AI self-efficacy mediates the relationship between organizational AI adoption and creativity.

### 2.4 The moderating role of empowering leadership

Self-determination theory suggests that external social contexts influence how individuals interpret and respond to technological change and work demands by supporting or thwarting their basic psychological needs, thereby shaping their motivation and subsequent behavior in organizations (Ryan and Deci, 2020; Gagné et al., 2022). In organizational settings, when employees perceive greater autonomy support, trust in their abilities, and resource support, their intrinsic motivation is more likely to be activated, making them more likely to show creative performance in active exploration and problem solving (Ye et al., 2025). Empowering leadership refers to leaders’ behaviors that enhance employees’ sense of autonomy and competence by giving them work autonomy, encouraging their participation in decision-making, and helping them understand the meaning of their work (Cheong et al., 2019).

Based on self-determination theory, empowering leadership, as an autonomy-supportive work context, can weaken the positive relationship between organizational AI adoption and employees’ AI replacement anxiety, and further alleviate its inhibitory effect on creativity. First, when empowering leadership is high, leader encourages employees to participate in AI-related work arrangements and technology-use decisions, giving them greater autonomy and involvement in the process of AI adoption, and reducing replacement anxiety caused by a loss of control (Cheong et al., 2019). Second, through information sharing, open communication, and emotional support, empowering leadership can reduce employees’ uncertainty about the purpose of organizational AI adoption, job adjustments, and future career changes, alleviating their threat perception of AI replacement risks (Lee et al., 2018). By contrast, under the condition of low empowering leadership, employees are more likely to interpret AI adoption as a signal of organizational monitoring, performance pressure, or job reduction, which may lead to stronger AI replacement anxiety (Yam et al., 2023). In addition, high empowering leadership may weaken the negative indirect effect of organizational AI adoption on employee creativity through AI replacement anxiety, whereas low empowering leadership may strengthen this negative indirect effect (Tong et al., 2025). Therefore, we propose:

*H7*: Empowering leadership negatively moderates the relationship between organizational AI adoption and AI replacement anxiety; that is, the higher the level of empowering leadership, the weaker the positive relationship between them.

*H8*: Empowering leadership negatively moderates the indirect relationship between organizational AI adoption and creativity through AI replacement anxiety; that is, the higher the level of empowering leadership, the weaker the negative effect of this indirect path.

Meanwhile, empowering leadership may strengthen the positive effect of organizational AI adoption on AI self-efficacy by enhancing the satisfaction of employees’ basic psychological needs, and further promote employee creativity. First, by granting employees greater decision-making authority, empowering leadership encourages them to explore and experiment independently when using AI technology, which satisfies their needs for autonomy and competence and makes the skill improvement brought by AI adoption more likely to develop into higher self-efficacy (Wörtler et al., 2022). Second, high empowering leadership can increase employees’ opportunities to engage with, learn, and skillfully use AI by expressing trust in their abilities, providing AI learning resources, and allowing trial and error in AI use, enabling them to accumulate successful experiences through continued practice and develop stronger AI self-efficacy (Kim et al., 2018; Knevelsrud et al., 2025). Moreover, related research also suggests that supportive leadership contexts can strengthen the extent to which technology-enabled resources foster efficacy beliefs and, in turn, contribute to employee creativity (Jeong and Jeong, 2024). Thus, we propose:

*H9*: Empowering leadership positively moderates the relationship between organizational AI adoption and AI self-efficacy; that is, the higher the level of empowering leadership, the stronger the positive relationship between them.

*H10*: Empowering leadership positively moderates the indirect relationship between organizational AI adoption and creativity through AI self-efficacy; that is, the higher the level of empowering leadership, the stronger the positive effect of this indirect path.

## 3 Methods

### 3.1 Procedure and research sample

This study used a two-wave time-lagged design to collect data in order to reduce the possible influence of common method bias. The research team contacted the human resource departments of several companies and conducted on-site questionnaire surveys after obtaining their support. In the first wave, the survey collected employees’ demographic information, including gender, age, education level, organizational tenure, monthly income, industry type and AI usage experience; and employees were also asked to rate organizational AI adoption and empowering leadership. During the survey, the research team visited the companies and organized employees to complete the questionnaires collectively. Respondents were informed of the research purpose and were told that the questionnaire was confidential and used only for academic research. A total of 460 questionnaires were distributed in this wave, and 432 were returned. One month later, the second-wave survey was conducted among employees who had completed the first-wave survey. The last four digits of respondents’ mobile phone numbers were used to match the two waves of data. In the second wave, employees rated AI replacement anxiety, AI self-efficacy, and creativity. After matching the questionnaires and screening the data, samples with missing values, obvious patterned responses, or failed matching were removed. Finally, 400 valid matched questionnaires were obtained, with an effective response rate of 86.96%.

The descriptive statistics of the final valid sample are as follows: 217 respondents (54.3%) were female, 145 respondents (36.3%) were aged 26–35, 169 respondents (42.3%) held a bachelor’s degree, 128 respondents (32.0%) had an organizational tenure of 7–12 months, and 146 respondents (36.5%) had a monthly income of RMB 7,000–8,999, 62 respondents (15.5%) worked in the service industry, and 124 respondents (31.0%) had 7–12 months of AI experience.

### 3.2 Measures

The variables in this study were measured using well-established scales from prior studies. To ensure that the scales were suitable for the present research context, this study used translation and back-translation procedures, and invited researchers in relevant fields to review the wording of the items to improve measurement accuracy. All items were measured on a five-point Likert scale, ranging from 1 = “strongly disagree” to 5 = “strongly agree.”

Organizational AI adoption. Organizational AI adoption was measured using the three-item scale developed by Cheng et al. (2023). A sample item is “My company has been involved in the adoption of AI technology” The Cronbach’s *α* was 0.873.

Employee creativity. Employee creativity was measured using the four-item scale developed by Farmer et al. (2003). A sample item is “I seek new ideas and ways to solve problems.” The Cronbach’s α was 0.906.

AI replacement anxiety. AI replacement anxiety was measured using the six-item scale developed by Wang and Wang (2022). A sample item is “I am afraid that an AI technique may replace humans.” The Cronbach’s α was 0.925.

AI self-efficacy. AI self-efficacy was measured using the three-item scale developed by Dong et al. (2025). A sample item is “I have mastered the skills necessary for my job related to the AI.” The Cronbach’s α of this scale was 0.863.

Empowering leadership. Empowering leadership was measured using the twelve-item scale developed by Ahearne et al. (2005). A sample item is “My manager makes many decisions together with me.” The Cronbach’s α of this scale was 0.924.

Control variables. This study included gender, age, education level, organizational tenure, monthly income, industry type, and AI usage experience as control variables.

## 4 Results

### 4.1 Reliability and construct validity

To assess the measurement model and construct validity, we used AMOS 24.0 to conduct confirmatory factor analysis on five latent variables. As shown in Table 1, the five-factor model showed the best fit compared with other alternative models (χ^2^ = 608.307, df = 340, χ^2^/df = 1.789, CFI = 0.960, TLI = 0.955, RMSEA = 0.044, SRMR = 0.038), indicating the five core variables in this study had good discriminant validity. We further examined the standardized factor loadings, CR, and AVE to assess the measurement properties of the constructs. As shown in Table 2, all standardized factor loadings were statistically significant and ranged from 0.653 to 0.906. The CR values ranged from 0.864 to 0.924, and the AVE values ranged from 0.503 to 0.709. These results provided further support for the internal consistency and convergent validity of the measurement scales. In addition, discriminant validity was assessed using the HTMT, all HTMT values ranged from 0.051 to 0.411 and were below the adopted threshold of 0.85, providing further evidence that the five constructs were empirically distinct.

**Table 1:** Results of CFA.

Model	χ^2^	df	χ^2^/df	CFI	TLI	RMSEA	SRMR
Five-factor model (OAA, EC, AA, AE, EL)	608.307	340	1.789	0.960	0.955	0.044	0.038
Four-factor model (OAA, EC, AA+AE, EL)	1287.071	344	3.741	0.859	0.845	0.083	0.095
Three-factor model (OAA + AA+AE, EC, EL)	1826.194	347	5.263	0.778	0.759	0.103	0.109
Two-factor model (OAA + AA+AE + EC, EL)	2779.536	349	7.964	0.636	0.606	0.132	0.132
One-factor model (OAA + AA+AE + EC + EL)	4716.252	350	13.475	0.346	0.294	0.177	0.302

OAA, Organizational AI adoption; EC, Employee creativity; AA, AI replacement anxiety; AE, AI self-efficacy; EL, Empowering leadership.

**Table 2:** Results of confirmatory factor analysis and reliability analysis.

Variables		Estimates	S.E.	C.R.	*p*	Standardized regression weights	SMC	CR	AVE
OAA	OAA3	1				0.774	0.599	0.896	0.683
	OAA2	1.120	0.064	17.468	***	0.849	0.721		
	OAA1	1.123	0.063	17.941	***	0.886	0.785		
AA	AA1	1				0.792	0.627		
	AA2	0.990	0.056	17.679	***	0.800	0.640	0.915	0.684
	AA3	1.077	0.057	18.953	***	0.844	0.712		
	AA4	0.996	0.056	17.941	***	0.809	0.654		
	AA5	1.091	0.056	19.392	***	0.858	0.736		
	AA6	1.012	0.055	18.314	***	0.822	0.676		
AE	AE1	1				0.814	0.663	0.864	0.679
	AE2	1.023	0.058	17.531	***	0.842	0.709		
	AE3	0.967	0.057	17.083	***	0.815	0.664		
EC	EC1	1.000				0.859	0.738	0.907	0.709
	EC2	1.101	0.047	23.548	***	0.906	0.821		
	EC3	0.918	0.049	18.692	***	0.780	0.608		
	EC4	0.963	0.048	20.123	***	0.817	0.667		
EL	EL12	1				0.695	0.483	0.924	0.503
	EL11	1.017	0.075	13.571	***	0.725	0.526		
	EL10	0.923	0.075	12.290	***	0.653	0.426		
	EL9	1.046	0.077	13.556	***	0.724	0.524		
	EL8	0.974	0.076	12.837	***	0.684	0.468		
	EL7	1.029	0.077	13.391	***	0.715	0.511		
	EL6	1.061	0.078	13.614	***	0.727	0.529		
	EL5	1.113	0.079	14.074	***	0.753	0.567		
	EL4	1.089	0.079	13.706	***	0.733	0.537		
	EL3	1.028	0.076	13.554	***	0.724	0.524		
	EL2	0.978	0.077	12.739	***	0.678	0.460		
	EL1	1.020	0.078	13.060	***	0.696	0.484		

### 4.2 Descriptive statistics and correlation analysis

Table 3 presents the means, standard deviations, and correlation coefficients of the study variables. The results showed that organizational AI adoption was positively correlated with both AI replacement anxiety (*r* = 0.369, *p* < 0.01) and AI self-efficacy (*r* = 0.352, *p* < 0.01). AI replacement anxiety was negatively correlated with creativity (*r* = −0.289, *p* < 0.01), while AI self-efficacy was positively correlated with creativity (*r* = 0.368, *p* < 0.01).

**Table 3:** Descriptive statistics and correlations for study variables.

Variable	1	2	3	4	5	6	7	8	9	10	11	12
1 Gender	—											
2 Age	−0.059	—										
3 Education	0.014	0.090	—									
4 Tenure	−0.027	−0.038	−0.025	—								
5 Income	−0.110^*^	−0.009	−0.053	−0.045	—							
6 Industry type	0.001	0.000	0.000	−0.002	0.000	—						
7 AI usage experience	0.002	0.003	0.004	−0.004	0.001	0.000	—					
8 OAA	0.054	0.011	0.001	−0.007	0.002	−0.093	−0.022	—				
9 AA	−0.057	−0.066	−0.126^*^	0.034	0.007	−0.064	0.036	0.369^**^	—			
10 AE	−0.037	−0.049	−0.009	−0.057	0.036	−0.029	−0.039	0.352^**^	−0.095	—		
11 EL	−0.028	−0.023	−0.011	0.044	0.038	0.008	−0.056	−0.001	−0.007	−0.001	—	
12 EC	−0.052	0.027	0.120^*^	−0.004	0.006	0.030	0.000	0.027	−0.289^**^	0.368^**^	0.011	—
M	0.540	2.280	2.740	2.670	2.760	3.940	2.390	3.008	3.045	3.034	3.120	3.033
SD	0.499	0.909	0.895	1.007	0.952	1.971	1.040	1.073	1.030	1.065	0.851	1.074

^*^p < 0.05, ^**^p < 0.01. Gender: Male (0), female (1); Age: ≤ 25 years (1), 26–35 years (2), 36–45 years (3), ≥ 46 years (4); Education: High school and below (1), junior college (2), bachelor’s degree (3), postgraduate degree or above (4); Tenure: ≤ 6 months (1), 7–12 months (2), 13–24 months (3), ≥25 months (4); Income: ≤5,000 yuan (1), 5,001–6,999 yuan (2), 7,000–8,999 yuan (3), ≥ 9,000 yuan (4). Industry type: construction (1), catering service(2), finance(3), education(4), medical(5), information technology(6), others(7); AI usage experience: ≤ 6 months (1), 7–12 months (2), 13–24 months (3), ≥25 months (4).

### 4.3 Hypothesis testing

To test the research hypotheses, this study used hierarchical regression analysis. After controlling for gender, age, education level, organizational tenure, income, industry type and AI usage experience, the relationships among the variables were examined. The results are shown in Table 4. Organizational AI adoption was significantly positively associated with AI replacement anxiety (M3, *β* = 0.373, *p* < 0.001) and AI self-efficacy (M6, *β* = 0.355, *p* < 0.001), supporting H1 and H4. AI replacement anxiety was significantly negatively associated with creativity (M9, *β* = −0.247, *p* < 0.001), H2 was supported. AI self-efficacy was significantly positively associated with creativity (M9, *β* = 0.347, *p* < 0.001), H5 was supported.

**Table 4:** Results of hierarchical linear analyses.

Variable	AA											
	M1				M2				M3			
	*β*	SE	*t*	*p*	*β*	SE	*t*	*p*	*β*	SE	*t*	*p*
Gender	−0.059	0.104	−1.166	0.245	−0.079	0.096	−1.700	0.090	−0.083	0.095	−1.798	0.073
Age	−0.057	0.057	−1.144	0.253	−0.063	0.053	−1.351	0.177	−0.070	0.052	−1.524	0.128
Education	−0.120	0.058	−2.400	0.017	−0.120	0.053	−2.585	0.010	−0.118	0.053	−2.567	0.011
Tenure	0.027	0.051	0.539	0.591	0.029	0.047	0.617	0.537	0.025	0.047	0.543	0.587
Income	−0.005	0.054	−0.098	0.922	−0.008	0.050	−0.171	0.864	−0.006	0.050	−0.130	0.896
Industry type	−0.064	0.026	−1.290	0.198	−0.029	0.024	−0.636	0.525	−0.024	0.024	−0.519	0.604
AI usage experience	0.037	0.049	0.741	0.459	0.045	0.046	0.981	0.327	0.042	0.045	0.925	0.355
OAA					0.373^***^	0.045	8.030	0.000	0.373^***^	0.044	8.142	0.000
AA												
AE												
EL									−0.019	0.055	−0.418	0.676
OAA × EL									−0.167^***^	0.051	−3.650	0.000
R^2^	0.029				0.166				0.194			
∆R^2^					0.137				0.165			
F	1.650				9.737^***^				9.353^***^			

Variable	AE											
	M4				M5				M6			
	*β*	SE	*t*	*p*	*β*	SE	*t*	*p*	*β*	SE	*t*	*p*
Gender	−0.038	0.108	−0.750	0.454	−0.058	0.101	−1.216	0.225	−0.055	0.100	−1.170	0.243
Age	−0.053	0.059	−1.052	0.293	−0.058	0.055	−1.234	0.218	−0.052	0.055	−1.118	0.264
Education	−0.003	0.060	−0.067	0.947	−0.003	0.056	−0.070	0.945	−0.006	0.056	−0.118	0.906
Tenure	−0.059	0.053	−1.177	0.240	−0.058	0.050	−1.222	0.222	−0.054	0.049	−1.151	0.250
Income	0.029	0.057	0.569	0.570	0.026	0.053	0.547	0.585	0.025	0.053	0.525	0.600
Industry type	−0.029	0.027	−0.569	0.570	0.004	0.026	0.094	0.925	0.000	0.025	−0.009	0.993
AI usage year	−0.039	0.051	−0.781	0.435	−0.031	0.048	−0.664	0.507	−0.029	0.048	−0.626	0.532
OAA					0.355^***^	0.047	7.496	0.000	0.355^***^	0.047	7.571	0.000
AA												
AE												
EL									0.005	0.059	0.103	0.918
OAA × EL									0.145^**^	0.054	3.099	0.002
R^2^	0.011				0.135				0.156			
∆R^2^					0.124				0.145			
F	0.614				7.636^***^				7.188^***^			

Variable	EC											
	M7				M8				M9			
	*β*	SE	*t*	*p*	*β*	SE	*t*	*p*	*β*	SE	*t*	*p*
Gender	−0.052	0.109	−1.030	0.304	−0.054	0.109	−1.064	0.288	−0.053	0.099	−1.167	0.244
Age	0.013	0.060	0.256	0.798	0.012	0.060	0.246	0.806	0.017	0.054	0.376	0.707
Education	0.120	0.060	2.390	0.017	0.120	0.060	2.388	0.017	0.092	0.055	2.010	0.045
Tenure	−0.002	0.054	−0.030	0.976	−0.001	0.054	−0.027	0.978	0.026	0.048	0.569	0.570
Income	0.007	0.057	0.131	0.896	0.006	0.057	0.126	0.900	−0.005	0.051	−0.101	0.919
Industry type	0.030	0.027	0.605	0.546	0.033	0.027	0.662	0.508	0.024	0.025	0.542	0.588
AI usage year	−0.001	0.052	−0.011	0.991	0.000	0.052	0.004	0.997	0.022	0.047	0.494	0.622
OAA					0.033	0.050	0.651	0.516	0.002	0.054	0.038	0.969
AA									−0.247^***^	0.053	−4.839	0.000
AE									0.347^***^	0.051	6.908	0.000
EL												
OAA × EL												
R^2^	0.018				0.020				0.214			
∆R^2^					0.002				0.196			
F	1.054				0.974				10.600^***^			

^***^p < 0.001; OAA and EL were mean-centered before the interaction term was constructed.

To further test the mediating effects of AI replacement anxiety and AI self-efficacy, this study used the PROCESS macro with the Bootstrap method. The indirect effect of organizational AI adoption on employee creativity through AI replacement anxiety was −0.092, with a 95% confidence interval of [−0.138, −0.052], excluding 0, thus H3 was supported. The indirect effect of organizational AI adoption on employee creativity through AI self-efficacy was 0.123, with a 95% confidence interval of [0.078, 0.174], excluding 0; H6 was supported. Importantly, these two indirect effects have opposite directions of action and similar magnitudes of effect, which may lead to the cancellation of the impact of organizational AI adoption on creativity.

**Figure 2:**
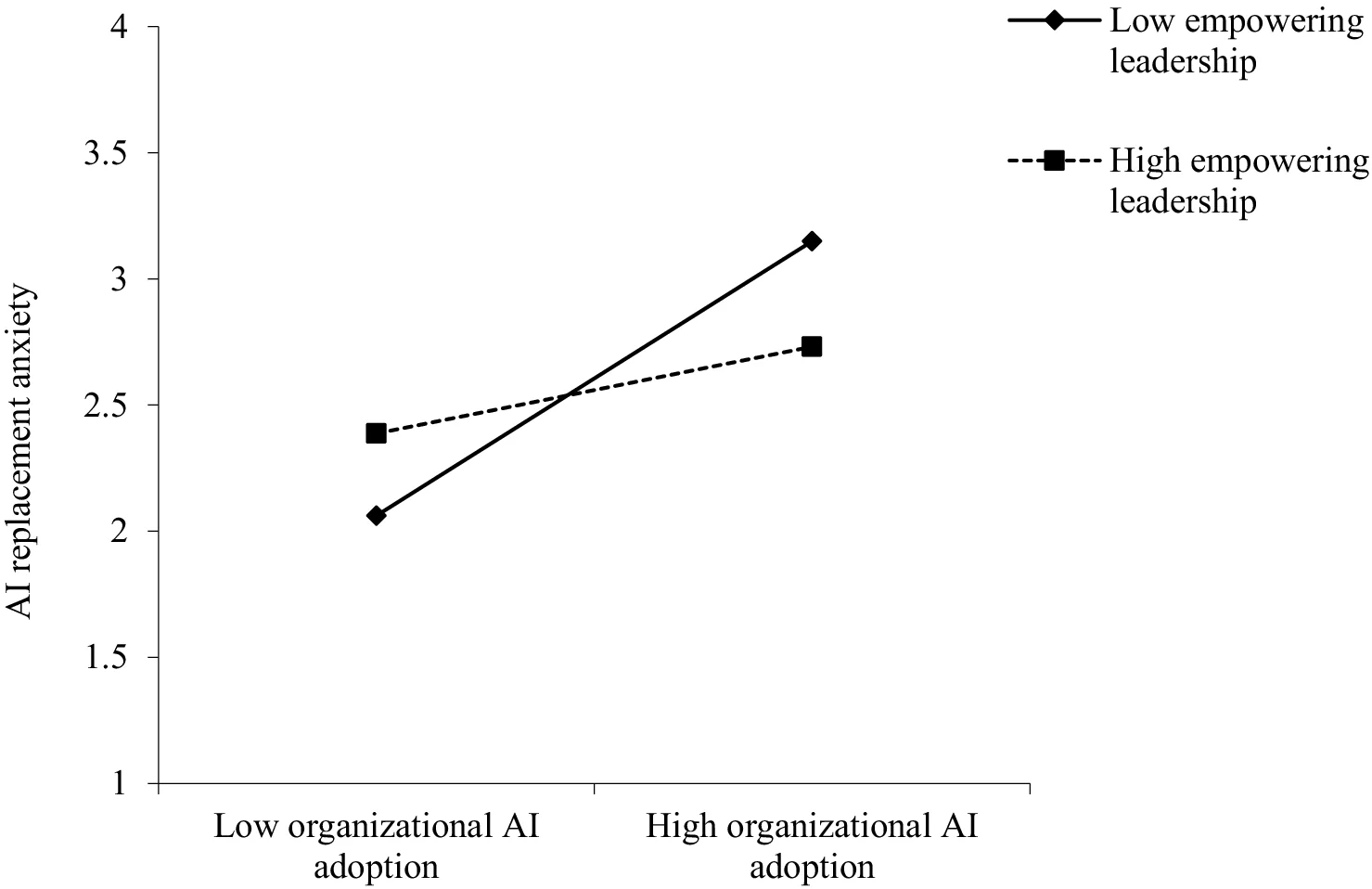
The moderating effect of empowering leadership on organizational AI adoption and AI replacement anxiety (simple slope plot).

**Figure 3:**
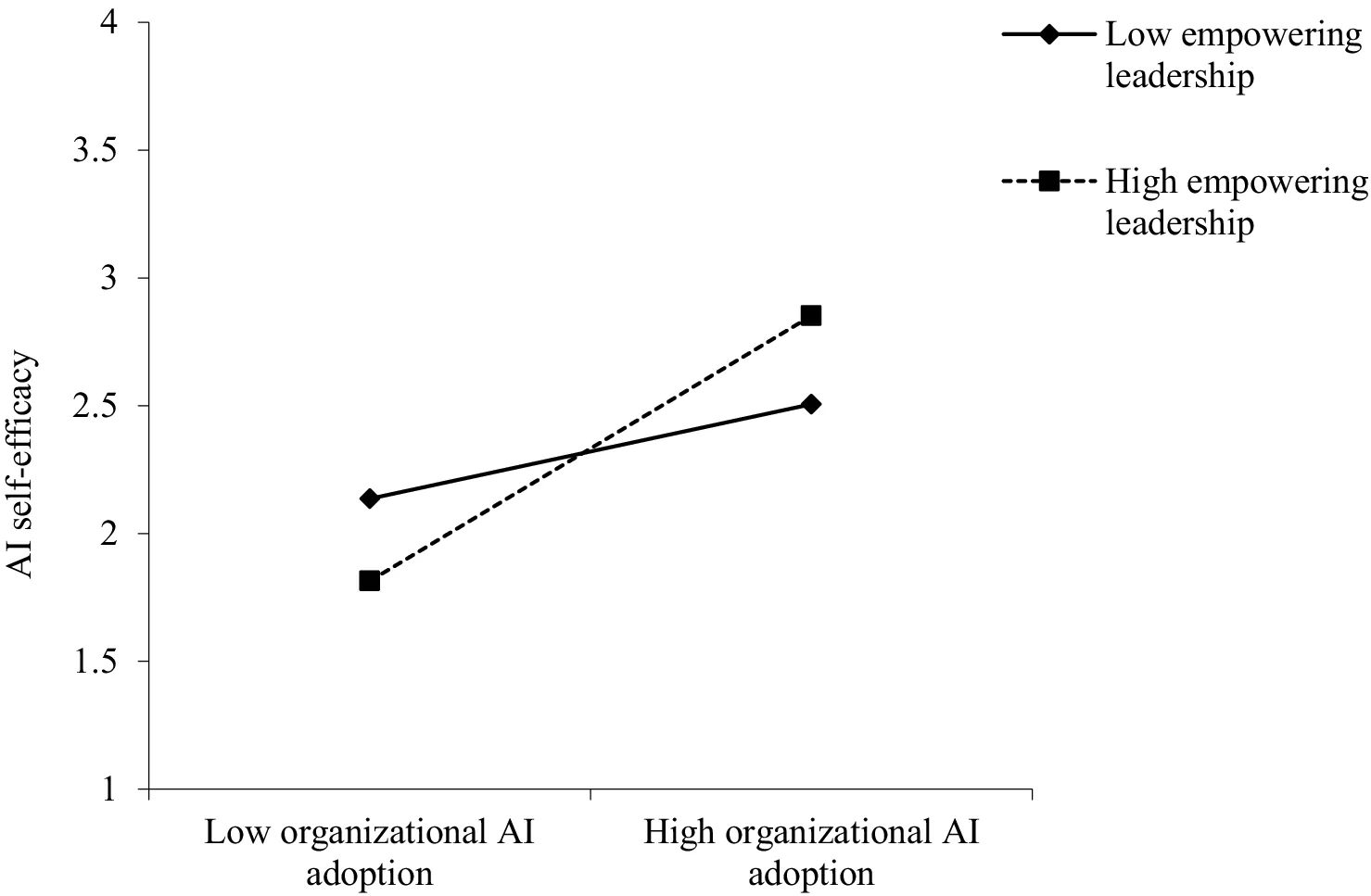
The moderating effect of empowering leadership on organizational AI adoption and AI self-efficacy (simple slope plot).

In addition, the interaction between organizational AI adoption and empowering leadership was negatively associated with AI replacement anxiety (M3, *β* = −0.167, *p* < 0.001) and was positively associated with AI self-efficacy (M6, *β* = 0.145, *p* < 0.001), supporting H7 and H9. We further plotted simple slope graphs, as shown in Figures 2, 3. Under low empowering leadership, the positive relationship between organizational AI adoption and AI replacement anxiety was stronger (*β* = 0.544, *p* < 0.001), whereas under high empowering leadership, the relationship between organizational AI adoption and AI replacement anxiety was weaker (*β* = 0.172, *p* < 0.05). Similarly, under low empowering leadership, the relationship between organizational AI adoption and AI self-efficacy was weaker (*β* = 0.185, *p* < 0.01), whereas under high empowering leadership, the positive relationship between organizational AI adoption and AI self-efficacy became stronger (*β* = 0.519, *p* < 0.001).

Moreover, to examine whether empowering leadership moderates the indirect effect of organizational AI adoption on employee creativity, this study conducted a moderated mediation analysis. As shown in Table 5, at a low level of empowering leadership, the indirect effect of organizational AI adoption on employee creativity through AI replacement anxiety was −0.133, with a 95% CI of [−0.195, −0.076]. While at a high level of empowering leadership, this indirect effect decreased to −0.052, with a 95% CI of [−0.091, −0.019]. The index of moderated mediation was 0.048, with a 95% CI of [0.020, 0.082], supporting H8. Similarly, at a low level of empowering leadership, the indirect effect of organizational AI adoption on creativity through AI self-efficacy was 0.074, with a 95% CI of [0.028, 0.125], while at a high level of empowering leadership, this indirect effect increased to 0.173, with a 95% CI of [0.111, 0.244]. The index of moderated mediation was 0.058, with a 95% CI of [0.022, 0.100], supporting H10.

**Table 5:** Moderated mediation effect test results.

Mediator	Moderating variable grouping	Effect	SE	95% confidence interval
AI replacement anxiety	M-SD	−0.133	0.030	[−0.195, −0.076]
	M	−0.092	0.021	[−0.136, −0.053]
	M + SD	−0.052	0.018	[−0.091, −0.019]
	Index	0.048	0.016	[0.020, 0.082]
AI self-efficacy	M − SD	0.074	0.025	[0.028, 0.125]
	M	0.123	0.024	[0.078, 0.172]
	M + SD	0.173	0.034	[0.111, 0.244]
	Index	0.058	0.020	[0.022, 0.100]

## 5 Discussion

### 5.1 Conclusion

The main conclusions are as follows. First, organizational AI adoption is associated with employee creativity through two opposing indirect pathways: a negative indirect association through AI replacement anxiety and a positive indirect association through AI self-efficacy. The positive and negative effects are close, making organizational AI adoption have no significant impact on creativity. Second, empowering leadership negatively moderates the relationship between organizational AI adoption and AI replacement anxiety, and positively moderates the relationship between organizational AI adoption and AI self-efficacy. Finally, empowering leadership also moderates the indirect effects of organizational AI adoption on creativity through AI replacement anxiety and AI self-efficacy.

### 5.2 Theoretical contributions

First, this study enriches research on the outcomes of organizational AI adoption. Existing studies have mainly examined employee well-being, retention intention, and technology acceptance, which capture how employees adapt to AI-related change (Valtonen et al., 2025; Jiang et al., 2026). Creativity reflects whether employees can embed AI-enabled information and technological resources into their work and transform them into novel and useful outcomes (Lee et al., 2023). This study responds to calls for greater attention to employee innovation and value creation in the AI era (Haefner et al., 2021). More importantly, the study does not merely show that organizational AI adoption has both positive and negative consequences. The negative indirect association through AI replacement anxiety and the positive indirect association through AI self-efficacy operate in opposite directions and are similar in magnitude, helping explain why prior studies may report weak, inconsistent, or even nonsignificant relationships between organizational AI adoption and employee creativity.

Second, this study extends the understanding of the mechanisms through which organizational AI adoption affects employee creativity. Prior research has drawn on conservation of resources theory, job demands–resources model, and social cognitive theory to explain resource gains and losses, work demands and resources, or efficacy beliefs associated with AI (Lin et al., 2024; Chuang et al., 2025; Liu et al., 2025). However, these perspectives provide limited explanation for why the same context of organizational AI adoption may trigger different, or even opposing, psychological responses among employees (Cheng et al., 2023). Self-determination theory provides a unified explanation by focusing on whether the work environment supports or thwarts employees’ autonomy and competence. Organizational AI adoption may reduce perceived control and create uncertainty about capability value and occupational continuity, while also providing learning opportunities, intelligent assistance, and task support. Accordingly, we conceptualize AI replacement anxiety and AI self-efficacy as downstream psychological responses associated with need-thwarting and need-supportive processes, respectively, thereby explaining how the same AI work environment can simultaneously generate opposing pathways to employee creativity.

Finally, this study expands our understanding of the boundary conditions under which organizational AI adoption affects employee creativity. Existing studies on the boundary mechanisms of AI adoption and employee behavior have mainly focused on individual characteristics, technological features, or organizational technology readiness (Cai et al., 2026; Zhang et al., 2026). Although these studies help explain why employees respond differently to AI, they have paid relatively limited attention to leadership behavior (Zhao et al., 2025). Because organization-level AI decisions are translated into employees’ daily work through supervisors’ task arrangements, information sharing, resource provision, and feedback, immediate supervisors constitute the primary social context through which employees interpret organizational AI adoption (Makarius et al., 2020; Wang et al., 2025). Empowering leadership provides decision authority, participation, information, confidence, and developmental support, thereby weakening the AI replacement anxiety pathway while strengthening the AI self-efficacy pathway (Cheong et al., 2019). Thus, rather than treating empowering leadership as merely another moderator, this study identifies it as the proximal social context through which organizational AI adoption is translated into employees’ psychological experiences, thereby enriching contextual explanations of organizational AI adoption.

### 5.3 Practical implications

First, enterprises should view the impact of organizational AI adoption on employee creativity rationally and guard against its possible negative psychological consequences. The findings show that organizational AI adoption does not necessarily promote employee creativity. When employees see AI as a signal of job replacement or threatened career development, they tend to experience AI replacement anxiety, which may reduce their creative engagement. Therefore, when promoting AI applications, enterprises should not focus only on efficiency improvement and cost optimization, but should also pay close attention to employees’ perceptions of and psychological responses to AI. Specifically, enterprises could consider establishing transparent AI communication mechanisms and clearly explain the goals, scope, and boundaries of human–AI task division in a timely manner, which may help reduce uncertainty and replacement concerns caused by information asymmetry. At the same time, enterprises may strengthen their support systems for career development and skill transformation by providing AI skills training, upgrading job-related capabilities, and offering internal career development plans, thereby potentially enhancing employees’ expectations of and control over their future career development. In addition, enterprises could consider optimizing human–AI collaboration models and position AI as a supportive tool for decision assistance and work quality improvement, rather than as an execution tool that simply replaces employees. Such practices may help employees recognize their unique value in creative thinking, complex judgment, and innovation activities, and may help alleviate the negative effect of AI replacement anxiety on employee creativity.

Second, enterprises should make full use of the positive role of organizational AI adoption in promoting employee creativity, with a particular focus on strengthening employees’ AI self-efficacy so that AI may become a tool for stimulating creativity. The findings suggest that the technological advantages brought by organizational AI adoption do not automatically lead to higher creativity. AI can become an important resource for supporting innovative ideas only when employees believe that they can effectively understand, master, and use it. Therefore, when promoting AI applications, enterprises could consider developing systematic AI empowerment mechanisms and help employees gradually master the use and application logic of AI tools through tiered training, hands-on exercises, and project-based practice, which may improve their ability to use AI to solve practical problems. At the same time, enterprises may consider creating low-risk AI application environments and encourage employees to conduct small-scale experiments and innovation exploration at work, thereby providing opportunities for them to build confidence in using AI through successful experiences. In addition, enterprises may establish AI application case libraries and experience-sharing platforms to promote good practices and help employees improve their AI application ability by observing and learning from others’ successful experiences. By continuously enhancing employees’ sense of control and competence in using AI, enterprises can help them transform AI from a simple work tool into an innovation partner, and more effectively convert technological advantages into innovation resources and creative outcomes.

Third, enterprises could consider the potential role of empowering leadership in creating a supportive context for organizational AI adoption. The findings indicate that, within the Chinese organizational sample examined in this study, the relationships between organizational AI adoption and employees’ AI-related psychological responses varied according to the level of empowering leadership. Therefore, in daily management, leaders may consider giving employees more autonomy and allow them to explore ways of using AI according to task characteristics, rather than promoting AI use simply through instructions. At the same time, leaders could encourage employees to participate in designing team-level AI application rules and work processes, and to discuss the boundaries between AI and human work, which may help strengthen their sense of participation and control in AI-related change. In addition, leaders may seek to foster a team climate that tolerates trial and error, regard exploration and failure in AI use as important parts of learning and growth, and support and recognize employees’ attempts to use AI to improve their work methods. Through empowerment, participation, and tolerance for trial and error, empowering leadership may weaken the effect of organizational AI adoption on AI replacement anxiety, while strengthening its effect on AI self-efficacy, thereby helping AI adoption translate more effectively into employee creativity.

### 5.4 Limitations and research directions

This study still has several limitations that should be addressed in future research. First, all data were collected from employees in Chinese enterprises. Although the findings can reflect how organizational AI adoption influences employee creativity in the Chinese organizational context, countries and regions may differ in cultural values, labor relations, technology governance, and employees’ acceptance of AI (Barnes et al., 2024). In addition, organizations may vary in AI adoption maturity, task integration, and the level of AI autonomy. Future research could replicate the present findings across different countries, cultural settings, and organizational contexts, and further distinguish between auxiliary and autonomous AI, low-maturity and high-maturity AI, and low and high task-integrated AI to examine the generalizability and robustness of the findings.

Second, although this study adopted a two-wave design, which helps reduce common method bias, it remains limited in capturing the dynamic evolution of organizational AI adoption, employees’ psychological responses, and creativity. This issue is particularly important because AI technologies are developing rapidly, and employees’ perceptions of organizational AI adoption may change as they become more familiar with AI capabilities and accumulate usage experience. For example, in the initial stage of AI adoption, employees may be more concerned about job replacement and skill devaluation, leading to stronger AI replacement anxiety. However, during continued use and relatively stable implementation, employees may gradually view AI as a capability-enhancing resource, thereby developing stronger AI self-efficacy. Future research could adopt three-wave or multi-wave longitudinal designs, as well as experimental or quasi-experimental methods, to examine more rigorously the temporal ordering, causal relationships, and dynamic changes among the focal variables.

Third, although self-determination theory provided the overarching framework for the proposed dual-path model, this study did not directly measure employees’ satisfaction or frustration of the needs for autonomy, competence, and relatedness. Accordingly, AI replacement anxiety and AI self-efficacy should be understood as AI-specific downstream psychological responses associated with need-thwarting and need-supportive processes rather than as direct indicators of basic psychological needs. Future research could directly examine the sequential mechanisms through which organizational AI adoption influences basic psychological need satisfaction or frustration, which subsequently shapes AI replacement anxiety, AI self-efficacy, and creativity. Other theoretical perspectives, such as psychological contract theory and sensemaking theory, may also help explain how employees interpret and respond to AI-related changes in work roles, skill requirements, and employment relationships (Hermann et al., 2025; Kim and Lee, 2025).

Finally, employee creativity was measured using self-reports in this study. Future research could combine employee self-reports with supervisor ratings, coworker evaluations, the number of creative suggestions, or other objective indicators to reduce potential social desirability and common-source bias. Future studies could also measure and control employees’ digital competence, technology readiness, AI usage intensity, baseline AI self-efficacy, and baseline creativity, and use latent profile analysis or configurational analysis to identify different psychological response patterns among employees.

## Data Availability

The raw data supporting the conclusions of this article will be made available by the authors, without undue reservation.
